# Leptin‐induced migration and angiogenesis in rheumatoid arthritis is mediated by reactive oxygen species

**DOI:** 10.1002/2211-5463.12326

**Published:** 2017-11-06

**Authors:** Xiaotong Sun, Jing Wei, Yawei Tang, Bing Wang, Yan Zhang, Lei Shi, Jianping Guo, Fanlei Hu, Xia Li

**Affiliations:** ^1^ Department of Immunology College of Basic Medical Science Dalian Medical University Liaoning China; ^2^ Department of Rheumatology and Immunology The Second Affiliated Hospital of Dalian Medical University Liaoning China; ^3^ College of Basic Medical Science Dalian Medical University Liaoning China; ^4^ Department of Rheumatology and Immunology Peking University People's Hospital Beijing China

**Keywords:** angiogenesis, leptin, migration, reactive oxygen species, rheumatoid arthritis

## Abstract

Rheumatoid arthritis (RA) is a progressive autoimmune disease affecting the joints. In this study, we investigated the role of the pro‐angiogenic factor leptin in regulating reactive oxygen species (ROS) to promote cell migration and angiogenesis in RA. We showed that leptin triggered RA fibroblast‐like synoviocyte (FLS) migration by increased ROS expression. Additionally, leptin enhanced human umbilical vein endothelial cell (HUVEC) tube formation in a ROS/hypoxia‐inducible factor‐1α‐dependent manner, accompanied by increased production of vascular endothelial growth factor and interleukin (IL)‐6. We also revealed that antagonists of tumor necrosis factor, IL‐6 and IL‐1β down‐regulated ROS production of RA FLS induced by leptin, which subsequently attenuated RA FLS migration and HUVEC tube formation. These findings demonstrated that leptin might play an important role in RA FLS migration and HUVEC angiogenesis.

**Abbreviations**
CM: conditioned medium
DCFHDA: 2′,7′‐dichlorofluorescein diacetate
DPI: diphenyleneiodonium chloride
FACS: fluorescence‐activated cell sorting
FLS: fibroblast like synoviocyte
GAPDH: glyceraldehyde 3‐phosphate dehydrogenase
HIF‐1α: hypoxia‐inducible factor‐1α
HUVEC: human umbilical vein endothelial cell
IL‐1βR: IL‐1β receptor
IL‐6R: IL‐6 receptor
IL: interleukin
NAC: *N*‐acetyl‐l‐cysteine
RA: rheumatoid arthritis
ROS: reactive oxygen species
TNFR: tumor necrosis factor receptor
TNF: tumor necrosis factor
VEGF: vascular endothelial growth factor

Rheumatoid arthritis (RA) is a progressive autoimmune disease associated with synovial hyperplasia, pannus formation and synovial inflammation of multiple joints, which leads to disruption of joint cartilage, bone loss and the disease spreading into normal joints. Number of cell populations including T cells, B cells, macrophages and fibroblast‐like synoviocytes (FLSs) are activated in the pathogenesis of RA. However, FLSs are the primary effector cells of synovial hyperplasia and the main origin of inflammatory mediators resulting in the initiation and development as well as the joint destruction of RA 1.

Since synovial hyperplasia and pannus formation are involved in the unique clinical characteristics of RA, some correlative biological behaviors of FLSs thought to bear on the course of RA. Firstly, the abnormal migration of RA FLSs were found to be responsible for joint inflammation and destruction in RA 1. Eisinger *et al*. 2 have already addressed the migratory potential of FLSs that favors their shifting to a distant unaffected joint and then eventually to the majority of joints. Secondly, RA FLSs in particular represent a major effector in the invasive pannus. Angiogenesis is a key aspect in pannus formation, and was modulated by vascular endothelial growth factor (VEGF) as well as its receptor, which favored blood vessel formation and immersion of leukocytes into synovial tissues of unaffected joint 3. It has also been documented that the expression of hypoxia‐inducible factor (HIF)‐1α, a cardinal transcription factor of VEGF 4, was elevated in RA FLSs with the stimulation of interleukin (IL)‐1 or tumor necrosis factor (TNF)‐α 5, indicating the unique role of FLSs in generating and maintaining the pannus in RA. So it has become widely held that regulation of the migratory and angiogenic actions of FLSs might be a promising treatment strategy for RA.

There are many factors promoting the migration of FLSs, such as histamine 6, protein arginine methyltransferase 5 7, growth differentiation factor 9 8, Toll‐like receptor 2 9 and placental growth factor 10. Leptin, initially identified as a pro‐angiogenic factor 11, participate in numerous biological processes. The functional leptin receptor is expressed in many cells, including vascular smooth muscle cells, endothelial cells and T lymphocytes. Thus, leptin is known to have multiple effects in promoting vascular remodeling, atherosclerosis and, more recently, angiogenesis 12. It was also documented that leptin elevated VEGF expression and promoted angiogenesis in human chondrosarcoma cells 13. Gonzalez‐Perez *et al*. 14 provided evidence on leptin signaling in the modulation of VEGF promoting tumor angiogenesis, and focused on the idea that leptin could trigger angiogenesis, growth and survival of breast cancer cells. Meanwhile emerging evidence indicated that leptin could up‐regulate the secretion of inflammatory cytokines including TNF‐α, IL‐6 and IL‐12 in RA patients 15, 16, 17. Our previous work (M. Wang, J. Wei, H. Li, X. Ouyang, X. Sun, Y. Tang, B. Wang, X. Li, unpublished data) illustrated that concentrations of leptin, positively related to the disease activity, were elevated in the serum of RA patients. In this study, we concentrated on whether leptin plays a role on the migration of RA FLSs and angiogenesis in RA patients.

## Materials and methods

### Patients and tissue specimens

Rheumatoid arthritis FLSs were isolated by enzymatic digestion of synovial tissues obtained from eight RA patients (F/M 6/2, median age 54 (range: 38–61) years, median duration 7.5 (range: 3–16) years) undergoing total joint replacement surgery. The rheumatoid factors were positive in seven patients. Erythrocyte segmentation rate and anti‐cyclic citrullinated peptide antibody checked preoperatively had median values of 54 (range: 25–84) mm·h^−1^ and 55 (range: 35–136) U·mL^−1^, respectively. All RA patients in this study fulfilled the American College of Rheumatology 2009 criteria for RA.

The tissues were minced after washing with PBS and digested with type I collagenase (Sigma‐Aldrich, St Louis, MO, USA) in Dulbecco's modified Eagle's medium (DMEM) for 2 h at 37 °C in 5% CO_2_. The study was agreed by the ethics committee of the Second Hospital of Dalian Medical University.

### Cell culture and treatment

Rheumatoid arthritis FLSs were cultured in DMEM supplemented with 10% FBS (Thermo Fisher Scientific, Waltham, MA, USA), and cell lines were used from three to five passages in this experiment. Human umbilical vein endothelial cell (HUVECs) were cultured in M199 medium (Gibco). All cells were cultured in medium supplemented with penicillin (50 U·mL^−1^) and streptomycin (50 μg·mL^−1^) and maintained in an incubator with 5% CO_2_ at 37 °C.

In some experiments, RA FLSs were stimulated with leptin after being preincubated with 2‐methoxyestradiol (a HIF‐1α inhibitor; Selleck, Houston, TX, USA), *N*‐acetyl‐l‐cysteine (NAC; Sigma‐Aldrich) and diphenyleneiodonium chloride (DPI; Sigma‐Aldrich). After 24 h incubation, culture supernatants were measured by ELISA. In other experiments, RA FLSs were pretreated with block antibodies of tumor necrosis factor receptor (TNFR) 2, IL‐6 receptor (IL‐6R) and IL‐1β receptor (IL‐1βR) (R&D Systems, Minneapolis, MN, USA) before being incubated with leptin. Detection of RA FLS migration, reactive oxygen species (ROS) level and HUVEC tube formation was performed as described below.

### Quantitative RT‐PCR

Total RNA was extracted from cells from six RA patients using RNAiso Plus (Takara Bio, Dalian, China) according to the manufacturer's instructions. The primers (Takara Bio) used to amplify genes were as follows: for HIF‐1α: 5′‐CTCAAAGTCGGACAGCCTCA‐3′ (forward), 5′‐CCCTGCAGTAGGTTTCTGCT‐3′ (reverse); glyceraldehyde 3‐phosphate dehydrogenase (GAPDH): 5′‐TGACCACAGTCCATGCCATCAC‐3′ (forward), 5′‐CGCCTGCTTCACCACCTTCTT‐3′ (reverse). Gene expression was quantified relative to the expression of the housekeeping gene GAPDH and normalized to control by standard 2^−∆∆CT^ calculation.

### ELISA assay

Supernatants from eight patients' RA FLSs were used for ELISA. ELISA kits used for detecting the concentration of cytokines in the supernatants according to the manufacturers' instructions were as follows: VEGF, IL‐1β and TNF‐α ELISA kits from SenBeiJia Bio (Nanjing, China) and IL‐6 ELISA kit from BioLegend (San Diego, CA, USA).

### Quantification of ROS levels

2′,7′‐Dichlorofluorescein diacetate (DCFHDA; Sigma‐Aldrich), a membrane‐permeant probe, was used to detect intracellular ROS level. Six RA patients' cells were used for ROS detection. After the addition of leptin for 1 h, RA FLSs were incubated with DCFHDA in serum‐free medium at 37 °C for 30 min. After washing twice with PBS, mean fluorescence intensity was determined and analyzed with a flow cytometer (Accuri C6; BD Biosciences, San Jose, CA, USA) at an excitation wavelength of 488 nm and an emission wavelength of 538 nm. Cells were observed under a microscope at ×100 magnification.

### Scratch assay

RA FLSs from six patients were used for a scratch assay. RA FLSs were drawn into a six‐well plate after 24 h at a confluence of 80%. Next, scratches were made in each well using a 200 μL pipette tip and replaced with serum‐free DMEM. The widths of wound at 0 and 24 h were determined from images obtained with an inverted microscope (Olympus 1X71, Tokyo, Japan) and evaluated by measuring the distance of the wound region in the absence of cells.

### Transwell migration assay

Rheumatoid arthritis FLSs from six patients were used for a Transwell migration assay. Cell migration was performed by using Transwell chambers (8 μm pores; Corning, New York, USA) in 24‐well plates. RA FLSs were added into the upper chambers and the lower chambers were filled with DMEM containing 10% FBS as the chemoattractant. After 24 h incubation, unmigrated cells in the upper chamber were removed, and the migrated cells on the lower side of the membranes were immersed into methanol for 10 min and stained with 0.1% crystal violet. Images were taken using an inverted microscope. Migrated cells were counted from five random fields (×100 magnification).

### HUVEC tube formation assay

Supernatants from six RA patients treated with vehicle or leptin for 24 h were collected and stored at −20 °C before using. Next, growth factor reduced Matrigel (BD Biosciences) was added into 48‐well plates (100 μL each) for gelatinizing at 37 °C for 30 min. Serum‐starved HUVECs were removed and resuspended in a 1 : 1 mixture of RA FLS culture supernatants and 1% FBS DMEM (total 200 μL) and then plated on the top of the Matrigel at 37 °C. Tube formation was observed every hour after cell seeding. Representative photos were taken at 8 h with an inverted microscope (×100 magnification) and the number of lengthened tubes were quantified using imagej software (NIH, Bethesda, MD, USA).

### Flow cytometry analysis

Six RA patients' cells were used for HIF‐1α protein measurement. After RA FLSs were treated with leptin for 4 h, the cells were fixed and permeabilized, then stained using phycoerythrin‐conjugated anti‐HIF‐1α (R&D Systems). Mean fluorescence intensity was analyzed with a flow cytometer (Accuri C6; BD Biosciences).

### Statistics

spss V.17.0 (SPSS Inc., Chicago, IL, USA) was used for statistical analysis. Statistical analysis was carried out using Wilcoxon's signed‐rank test or one‐way ANOVA. A *P*‐value of < 0.05 was considered statistically significant.

## Results

### Leptin triggered RA FLS migration and promoted HUVEC tube formation

Rheumatoid arthritis FLSs have tumor‐like characteristics including migration and angiogenesis, which may be caused by chronic exposure to a variety of stimuli. Leptin has been reported to increase cell migration and angiogenesis in some types of cancer cells and myelomonocytic cells 18, 19. In this study, we wanted to know whether leptin can promote RA FLS migration and HUVEC tube formation.

**Figure 1:**
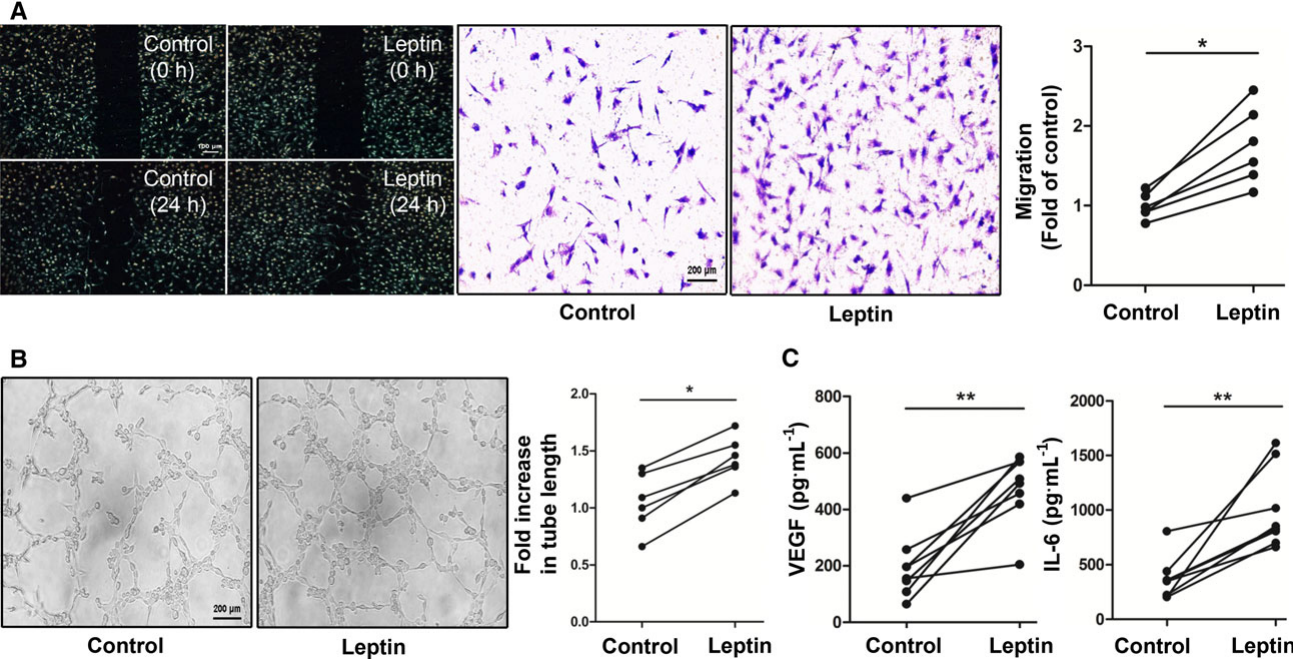
Leptin induced RA FLS migration and HUVEC tube formation. (A) RA FLSs isolated from RA patients were stimulated with or without leptin (100 ng·mL^−1^) for 24 h. Cell migration was measured by using the scratch assay and Transwell chambers. Representative photographs of control and leptin‐treated cells at 0 and 24 h are shown (*n* = 6). (B) RA FLSs were treated with or without leptin (100 ng·mL^−1^) for 24 h. CM was then collected and applied to HUVEC cultures after addition of these cells to the Matrigel. The number of HUVEC tubes formed was determined by microscopy (*n* = 6). (C) RA FLSs were stimulated with or without leptin (100 ng·mL^−1^) for 24 h. The level of VEGF and IL‐6 in the supernatant was determined by ELISA (*n* = 8). All experiments were repeated three times. Data represent the mean ± SEM (Wilcoxon's signed‐rank test; *P < 0.05, **P < 0.01).

First, we explored the influence of leptin on RA FLS migration. Scratch assays and Transwell migration assays were performed after leptin had stimulated RA FLSs for 24 h. As shown in the scratch assay, leptin‐treated RA FLSs were more likely to migrate into the created cell‐free area than untreated FLSs. Subsequently, FLS migration was determined by a Transwell migration assay. RA FLSs treated with leptin exhibited strong upregulation of migration compared with the control group (*P ≤ 0.05; Fig. 1A).

Angiogenesis has been considered to be a critical step in the initiation and progression of chronic arthritis 20. RA FLSs, as important inflammatory cells, can release proangiogenic growth factors including VEGF and IL‐6, which facilitate neovascularization. Here we observed tube formation of HUVECs that were treated with conditioned medium (CM) derived from leptin‐stimulated RA FLSs or untreated RA FLSs. As we expected, leptin‐treated CM induced much more tube formation than vehicle‐treated CM (*P ≤ 0.05; Fig. 1B). We also found that leptin‐stimulated RA FLSs had markedly increased levels of VEGF and IL‐6 in culture supernatants (**P ≤ 0.01; Fig. 1C).

### ROS production was involved in leptin‐induced RA FLS migration and HUVEC tube formation

**Figure 2:**
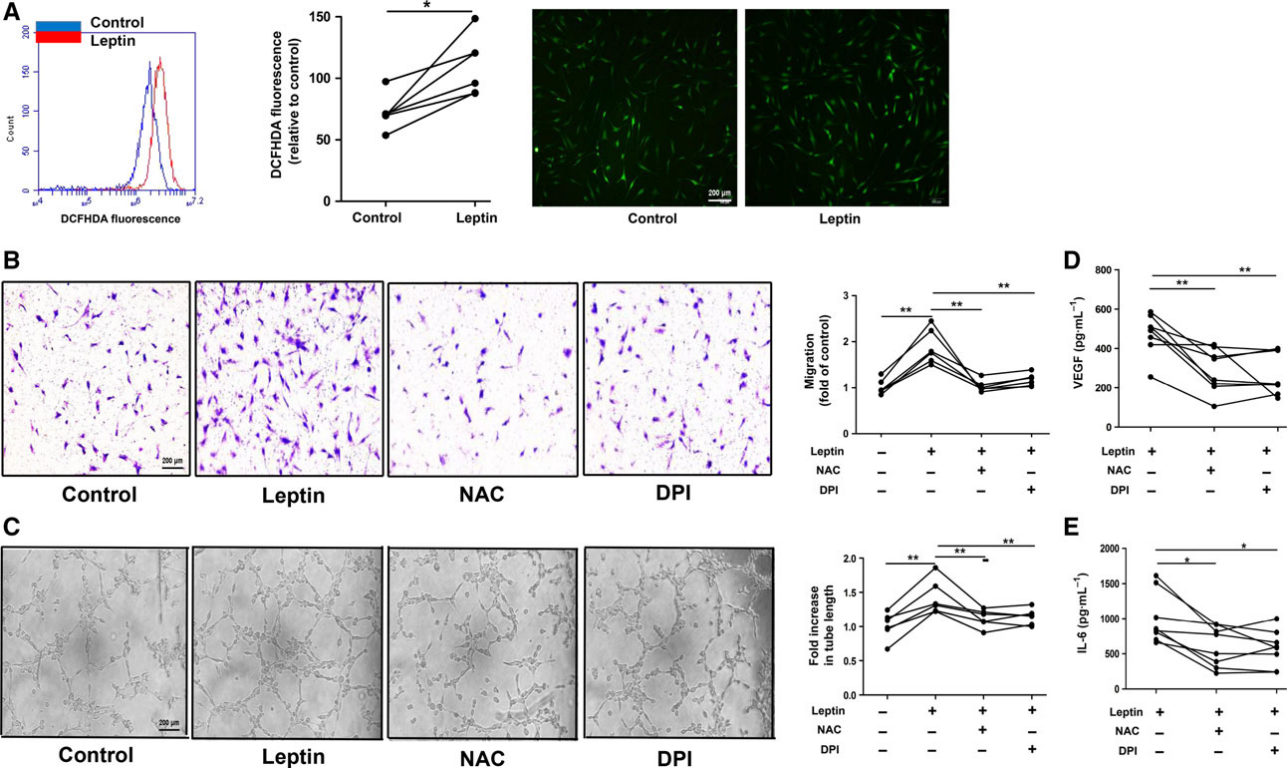
ROS generation was involved in leptin‐stimulated RA FLS migration and HUVEC tube formation. (A) RA FLSs were labelled with DCFHDA (5 μm) after being incubated with leptin (100 ng·mL^−1^) for 1 h. The fluorescent intensity of ROS was measured by flow cytometry and immunofluorescence (*n* = 6). (B,C) RA FLSs were pretreated with NAC (5 mm) or DPI (5 μm) for 1 h, and then stimulated with leptin (100 ng·mL^−1^) for 24 h. Cell migration was examined with Transwell chambers. Matrigel assay was performed to test HUVEC tube formation (*n* = 6). (D,E) The levels of VEGF and IL‐6 in the supernatant were measured by ELISA (*n* = 8). All experiments were repeated three times. Data represent the mean ± SEM (Wilcoxon's signed‐rank test; *P < 0.05, **P < 0.01).

Studies have shown that IL‐1β induces endothelial cell angiogenesis by upregulating fibroblast growth factor 2 accompanied with increased ROS production 21, which suggests that ROS might be related to the angiogenesis process. First, to evaluate the effects of leptin on ROS generation by RA FLSs, cells were incubated with or without leptin for 24 h, and the intracellular ROS level was determined with the DCFHDA fluorescent probe. The result showed a remarkable increase in DCFHDA fluorescence in leptin‐treated RA FLSs using fluorescence‐activated cell sorting (FACS) and immunofluorescence analysis (*P ≤ 0.05; Fig. 2A). Next, to determine whether ROS took part in leptin‐induced FLS migration and HUVEC tube formation, NAC (a ROS scavenger) and DPI (a ROS inhibitor) were used to block the effect of ROS. Pretreatment of RA FLS with NAC and DPI significantly attenuated leptin‐triggered RA FLS migration (**P ≤ 0.01; Fig. 2B). Furthermore, the tube formation stimulated by leptin‐treated CM was significantly inhibited by pretreatment with NAC and DPI (**P ≤ 0.01; Fig. 2C) and the levels of VEGF and IL‐6 were also decreased (*P ≤ 0.05, **P ≤ 0.01; Fig. 2D,E). The results suggest that ROS production of leptin‐treated RA FLSs was involved in RA FLS migration and HUVEC tube formation.

### ROS‐mediated leptin‐induced HUVEC tube formation via the activation of the HIF‐1α pathway

**Figure 3:**
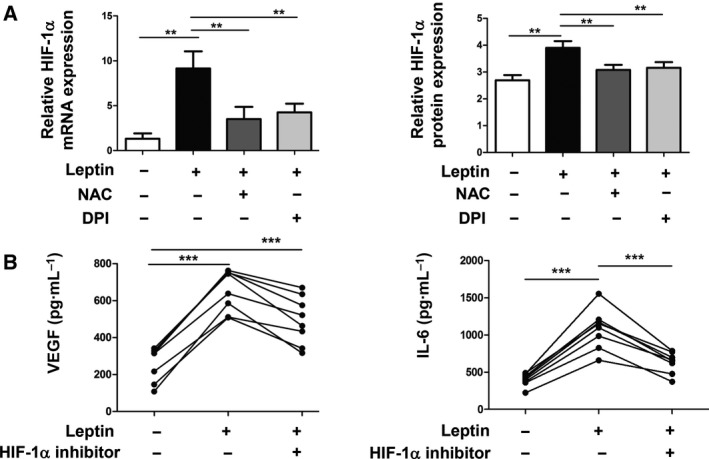
The ROS/HIF‐1α pathway participated in leptin‐induced HUVEC tube formation. (A) RA FLSs were pretreated with NAC and DPI for 1 h and then stimulated with leptin (100 ng·mL^−1^) for 4 h. HIF‐1α mRNA expression of RA FLSs was determined by real‐time PCR. GAPDH was used as a control in real‐time PCR. FACS was used to detect HIF‐1α protein level (*n* = 6). (B) RA FLSs were preincubated with 10 μm 2‐methoxyestradiol (a HIF‐1α inhibitor) for 1 h and then stimulated with leptin (100 ng·mL^−1^) for 24 h. The levels of VEGF and IL‐6 in the supernatant were examined by ELISA (*n* = 8). All experiments were repeated three times. Data represent the mean ± SEM (one‐way ANOVA; **P < 0.01, ***P < 0.001).

To further explore the mechanisms of leptin‐induced HUVEC tube formation, we also examined the activation of HIF‐1α, a related transcription factor that regulates VEGF expression by binding to hypoxia‐response element. First, we explored the effect of leptin on HIF‐1α expression of RA FLSs. The results from qPCR and FACS indicated that HIF‐1α mRNA expression and protein level were significantly increased in leptin‐treated RA FLSs (**P ≤ 0.01; Fig. 3A). Next, further analysis showed that leptin‐mediated HIF‐1α expression could be markedly abrogated by NAC and DPI (**P ≤ 0.01; Fig. 3A), which indicated that leptin promoted HIF‐1α expression on RA FLS via ROS production. Moreover, ELISA demonstrated that leptin‐induced VEGF and IL‐6 levels could be reduced by HIF‐1α inhibitor treatment (2‐methoxygestradiol; ***P ≤ 0.001; Fig 3B). These results indicated that leptin‐induced HIF‐1α expression might serve as the downstream effector of ROS, which promoted VEGF and IL‐6 production in RA FLSs.

### Effect of antagonists of TNFR2, IL‐6R and IL‐1βR on ROS generation, RA FLS migration and HUVEC tube formation

**Figure 4:**
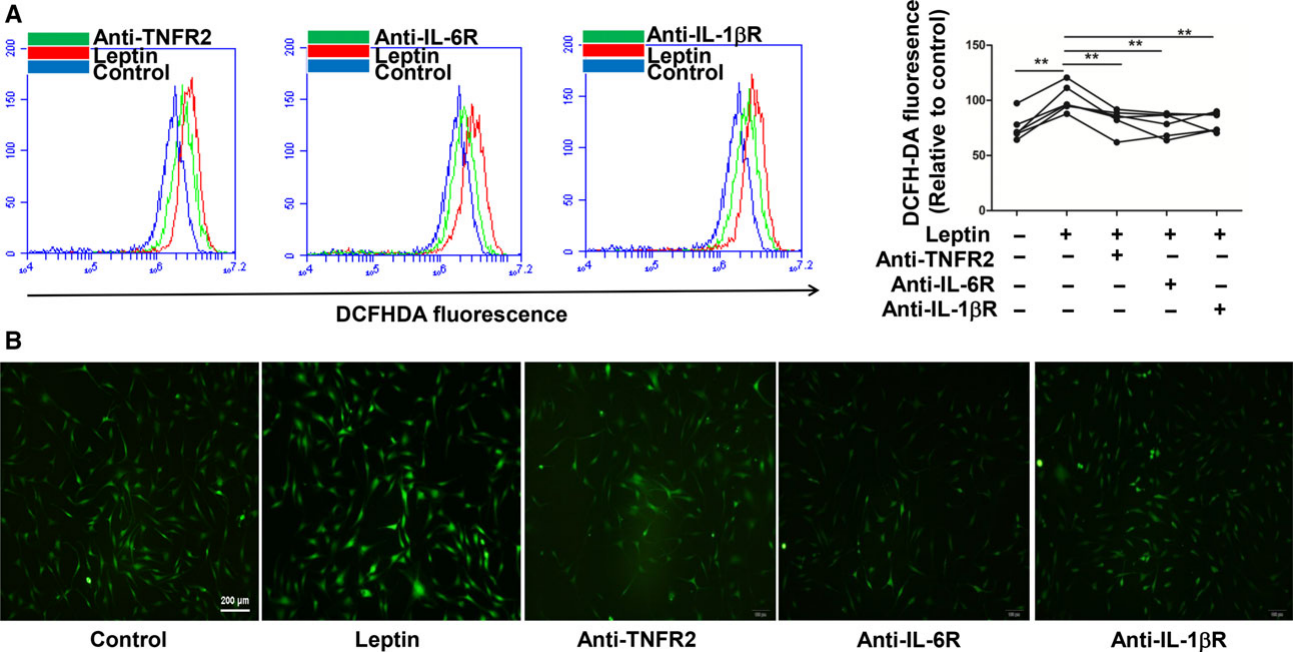
ROS production was decreased by antagonists of TNF, IL‐6 and IL‐1β. RA FLSs were incubated with anti‐TNFR2 (0.25 μg·mL^−1^), anti‐IL‐6R (0.5 μg·mL^−1^) and anti‐IL‐1βR (0.5 μg·mL^−1^) for 2 h and then cultured with leptin (100 ng·mL^−1^) for 1 h. ROS production was measured by flow cytometry (A) and immunofluorescence (B) (*n* = 6). All experiments were repeated three times. Data represent the mean ± SEM (one‐way ANOVA; **P < 0.01).

**Figure 5:**
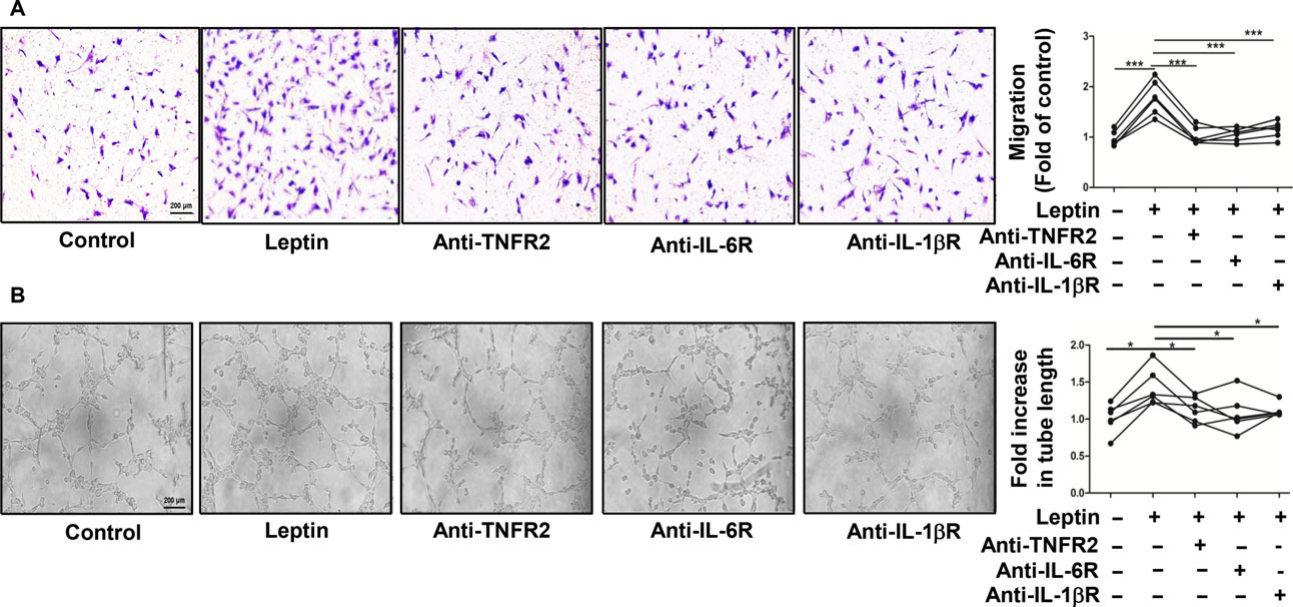
RA FLS migration and HUVEC tube formation were attenuated by antagonists of TNF, IL‐6 and IL‐1β. RA FLSs were pretreated with anti‐TNFR2 (0.25 μg·mL^−1^), anti‐IL‐6R (0.5 μg·mL^−1^) and anti‐IL‐1βR (0.5 μg·mL^−1^) for 2 h before stimulated with leptin (100 ng·mL^−1^) for 24 h. (A) RA FLS migration was tested by Transwell chambers (*n* = 6). (B) A Matrigel assay was performed to measure HUVEC tube formation by microscopy (*n* = 6). All experiments were repeated three times. Data represent the mean ± SEM (one‐way ANOVA; *P < 0.05, ***P < 0.001).

The blockades of TNF, IL‐6 and IL‐1 are widely used in clinical RA treatment and are beneficial in ameliorating disease and controlling symptoms. Can antagonists of these cytokines influence RA FLS migration and HUVEC tube formation by down‐regulating leptin‐induced ROS production? We preincubated anti‐TNFR2, anti‐IL‐6R and anti‐IL‐1βR with leptin‐stimulated RA FLSs separately. A decline in ROS generation (**P ≤ 0.01; Fig. 4A,B), RA FLS migration (***P ≤ 0.001; Fig. 5A) and HUVEC tube formation (*P ≤ 0.05; Fig. 5B) was discovered in treatments with antagonists of TNFR2, IL‐6R and IL‐1βR.

## Discussion

In the present study, we showed that leptin triggered RA FLS migration by increased ROS expression. Additionally, leptin enhanced HUVEC tube formation in a ROS/HIF‐1α‐dependent manner, accompanied by elevated VEGF and IL‐6 production. We also revealed that antagonists of TNFR, IL‐6R and IL‐1βR down‐regulated ROS production of RA FLSs induced by leptin, which subsequently attenuated RA FLS migration and HUVEC tube formation.

Rheumatoid arthritis is mainly characterized by synovial hyperplasia, activation of inflammatory cells and synovium invasion 22. RA FLSs, as key players in joint destruction and migration, have been shown to contribute to the formation of invasive pannus through migrating to locally adjacent joints susceptible to matrix destruction and even to distant unaffected joints through the bloodstream 23. Several inflammatory factors such as IL‐21 have been reported to promote RA FLS migration and invasion by upregulating matrix metalloproteinase in the phosphoinositide 3‐kinase–signal transducer and activator of transcription‐3 pathway 24. Angiogenesis is the outgrowth and proliferation of capillaries from pre‐existing blood vessels. In RA, the formation of new blood vessels provides oxygen and nutrients to the hypertrophic joint and supports the elevated transendothelial leukocyte infiltration that promotes synovial inflammation and bone destruction 20. During active disease, RA FLSs are triggered by inflammatory mediators to produce proangiogenic growth factors and cytokines such as VEGF and IL‐6. VEGF is a key regulator in the starting phase of angiogenesis, while IL‐6 plays a central role in RA angiogenesis through both its direct effect on endothelial cells and its indirect effect on different cell types in RA synovium to produce proangiogenic factors 22. Kayakabe *et al*. 25 proved that IL‐6 was capable of inducing VEGF production in a co‐culture system of RA FLSs and endothelial cells.

Leptin is one of the most important hormones secreted by adipose tissue and it regulates appetite, bone mass, basal metabolism, reproductive function and insulin secretion by binding and activating the long form of leptin receptor 26. Recent studies showed that leptin signaling increased cell proliferation in breast cancer, led to cells evading apoptosis and induced angiogenesis 13, 27, 28. The latest findings emphasize the role of leptin in the autoimmune and inflammatory rheumatic diseases, such as RA 29, 30. Plasma leptin levels correlated with 28‐joint disease activity score and IL‐17 in RA patients with conventional pharmacological treatment, which suggested that leptin could be a biomarker of long‐term disease 31. Our previous study showed that leptin could increase peripheral CD4^+^CXCR5^+^ICOS^+^ T cell numbers in RA patients (data not shown). In this study, we demonstrated that leptin promoted RA FLS migration and leptin‐treated RA FLS CM facilitated HUVEC capillary‐like structure formation, accompanied by the up‐regulation of VEGF and IL‐6 levels.

Hyperplasia of RA FLSs leads to over‐proliferation of synovial tissue, which results in increased oxygen consumption in synovium and thereby forms a hypoxic environment. Hypoxia is essential for the inflammation, angiogenesis and cartilage degradation of RA and is associated with the generation of ROS. Accumulating evidence has shown that excess production of ROS is associated with cell migration, inflammation and apoptosis. In the present study, we found leptin‐induced ROS expression markedly promoted RA FLS migration. However, the master regulators of the immune response to oxygen tension are HIFs, levels of which are sensitive to oxygen tension changes 32, 33. HIF‐1 is a heterodimeric transcription factor composed of HIF‐1α, which is oxygen‐regulated, and HIF‐1β, which is expressed constitutively in nuclei. A study has shown that the increase of cellular ROS generation amplified HIF‐1α stabilization and HIF‐dependent expression of genes such as that for VEGF 34. Here, we found that leptin‐stimulated HIF‐1α expression was mediated by ROS accompanied with increased VEGF and IL‐6 levels. Taken together, these findings indicated that HIF‐1α was a downstream regulator of leptin‐induced ROS production, which regulated angiogenesis via up‐regulating VEGF and IL‐6 expressions.

Inflammatory cytokines such as TNF‐α, IL‐6 and IL‐1 are important mediators in driving inflammation and joint destruction in RA. TNF and IL‐1 stimulate synoviocytes and chondrocytes to release matrix metalloproteinases and other proteinases, and up‐regulate the expression of proinflammatory genes, resulting in elevated production of various proinflammatory mediators 35, 36. IL‐6 promotes survival of B cells and their differentiation into autoantibody‐secreting long‐lived plasma cells leading to RA joint destruction 37. TNF, IL‐6 and IL‐1β blocking agents in RA patients resulted in a decline in disease severity and relief of arthritis symptoms. In this study, we have shown that antagonists of TNF, IL‐6 and IL‐1 could down‐regulate leptin‐induced ROS production and attenuate RA FLS migration and angiogenesis, which might offer a promising mechanism for clinical treatment of RA.

In summary, our findings clearly demonstrated that leptin induced RA FLS migration and promoted HUVEC tube formation through the activation of the ROS/HIF‐1α signaling pathway. This study suggested that leptin inhibition could be a potential therapeutic target for the prevention of joint invasion and angiogenesis in RA. However, the mechanisms of how leptin promoted RA FLS migration and angiogenesis need further exploration.

## Author contributions

XS and JW planned the experiments, performed the experiments, analyzed the data and wrote the manuscript. YT, YZ, BW, LS, JG and FH interpreted the experiments and analyzed the data. XL planned the experiments, analyzed data, modified the paper and approved the final version of the manuscript submitted for publication.
